# Energy-Efficient Object Detection and Tracking Framework for Wireless Sensor Network

**DOI:** 10.3390/s23020746

**Published:** 2023-01-09

**Authors:** Jayashree Dev, Jibitesh Mishra

**Affiliations:** 1 Department of Information Technology, Odisha University of Technology and Research, Bhubaneswar 751003, India; 2 Department of Computer Science & Application, Odisha University of Technology and Research, Bhubaneswar 751003, India

**Keywords:** wireless sensor network, energy efficiency, object detection, object tracking, object localization

## Abstract

Object detection and tracking is one of the key applications of wireless sensor networks (WSNs). The key issues associated with this application include network lifetime, object detection and localization accuracy. To ensure the high quality of the service, there should be a trade-off between energy efficiency and detection accuracy, which is challenging in a resource-constrained WSN. Most researchers have enhanced the application lifetime while achieving target detection accuracy at the cost of high node density. They neither considered the system cost nor the object localization accuracy. Some researchers focused on object detection accuracy while achieving energy efficiency by limiting the detection to a predefined target trajectory. In particular, some researchers only focused on node clustering and node scheduling for energy efficiency. In this study, we proposed a mobile object detection and tracking framework named the Energy Efficient Object Detection and Tracking Framework (EEODTF) for heterogeneous WSNs, which minimizes energy consumption during tracking while not affecting the object detection and localization accuracy. It focuses on achieving energy efficiency via node optimization, mobile node trajectory optimization, node clustering, data reporting optimization and detection optimization. We compared the performance of the EEODTF with the Energy Efficient Tracking and Localization of Object (EETLO) model and the Particle-Swarm-Optimization-based Energy Efficient Target Tracking Model (PSOEETTM). It was found that the EEODTF is more energy efficient than the EETLO and PSOEETTM models.

## 1. Introduction

One of the key applications of WSNs is object detection and tracking. Examples of this application include surveillance systems, smart home systems, smart cities, wildlife monitoring, environment monitoring, etc. Although WSN-based object detection and tracking systems are cost-effective, they face a number of challenges [1,2], such as sensor node failure, network coverage and connectivity, node cooperation, the recovery of targets, energy management, data aggregation, data transmission, the stability of the network, sensor technology, node localization techniques, reporting frequency, object localization precision, sampling frequency and the security of the network. There is often a chance of sensor node failure due to early battery depletion, a natural problem, or hardware failure. The loss of a target arises due to the presence of an obstacle in the AOI, low tracking precision or changes in the object speed and object path. Data aggregation at a cluster head may create unnecessary delays in transmitting messages to the BS. There are many reasons why early energy depletion limits the network lifetime, including large size overheads, very high node density, congestion, the number of transmissions, etc. Network coverage holes affect the tracking precision. In the past, many studies have been carried out to mitigate these issues and develop a robust object detection and tracking system. However, to date significant success has not been achieved. This is why today researchers have not lost any interest in this topic.

Three types of network structures are used for object detection and tracking: tree structures, cluster structures and flat structures. Among them, the cluster structure is the most commonly used network structure, as it guarantees a lower level of energy consumption by limiting the number of transmissions among nodes. The task of object detection and tracking mainly consists of two steps: the detection of an object’s movement path and the estimation of the exact location of the object at a particular period of time. Different types of object detection and tracking schemes include prediction-based tracking, probabilistic tracking, etc. Both of these schemes are used to implement sleep–awake schedules for deployed sensors. In prediction-based tracking, the next location of the object is predicted from the current location and past location. Based on the next location, nearby nodes are awakened for cooperation in tracking. In the case of probabilistic tracking, the probability of the appearance of a target at a particular location is calculated from the past data, and accordingly nearby nodes are awakened for tracking. To determine the object position at a particular time in the AOI, two methods are used: the range-based positioning method and the range-free positioning method. In the range-based method, GPS-equipped nodes are used for object location estimation, whereas in the range-free method a few GPS-based nodes, known as anchor nodes, are used. Range-free methods are popular as they are cheap, but the precision of the results is lower in comparison to range-based methods.

To be energy efficient, a typical target tracking scheme should consider the following points [3]:

- The selection of the optimum number of nodes for object detection and localization;
- A distributed prediction algorithm for the optimal prediction of an object’s state;
- The selection of the optimum data reporting path;
- The optimum node activation mechanism;
- The optimum logical network structure;
- The optimal node synchronization scheme.

In the past, researchers have studied object detection and tracking in homogeneous WSNs from different perspectives in order to mitigate the associated issues. Most researchers aimed to enhance the lifetime of a network while achieving object detection accuracy by deploying as many nodes as possible. They neither considered the cost of the system nor the accuracy of object localization. Some researchers have focused on the accuracy of object detection by tracking the object in a predefined object path while achieving energy efficiency by optimizing the mobile node trajectory. They neither considered the case of tracking an object at any place in the AOI nor accurate object localization. No researcher has previously focused on the case of robust data reporting for the minimization of information loss. Similarly, we can find a lot of work on smartphone-assisted mobile platforms. Though they provide improved quality service, they require sophisticated platforms for operation and are not cost effective.

In this paper, we proposed an energy-efficient object detection and tracking framework for heterogeneous WSNs called the EEODTF. The framework focuses on the issue of energy efficiency in WSN-based object detection and tracking while not affecting the accuracy of object detection and localization. The issue of energy efficiency is addressed by focusing on energy-efficient node deployment, energy-efficient data reporting or routing and energy-efficient object detection. The issue of object detection and localization accuracy is handled by maximizing the network coverage, minimizing the node localization error and minimizing the object detection error.

The contributions of this paper are:

- Energy-efficient node deployment for high network coverage and connectivity in order to achieve a high level of accuracy in object detection. Minimizing the number of hardware components will minimize the cost of the system.
- A node localization solution to minimize object localization errors.
- Energy-efficient object detection.
- Energy-efficient routing of object detection information to the BS.

In Section 2, a brief description of literature relating to our work is provided, and in Section 3, a description of the proposed object detection and tracking framework is presented. In Section 4, the simulation environment and results are described. In Section 5, conclusions are drawn, and information related to future work is provided.

## 2. Literature Review

In this section, we discuss the current key research that has been conducted regarding energy-efficient object detection and tracking.

J. Rejina Parvin and C. Vasanthanayaki [4] proposed a target tracking model named Particle-Swarm-Optimization-based Energy-Efficient Target Tracking (PSOEETTM) for wireless sensor networks, which focuses on distributed energy optimization by optimizing the mobile node movement while tracking the object. The model uses both static and mobile nodes for object tracking. Periodically, when the object is sensed, its next location is predicted based on its current position, velocity and angle of movement, and accordingly mobile node locations for the next time period are predicted, and mobile nodes are moved to those locations. The objective of moving mobile nodes is to cover the trajectory of the moving object in order to achieve a high level of detection accuracy. This causes the wastage of energy for the sake of accuracy. Again, the number of nodes to be deployed is not considered. It is assumed that high network coverage and connectivity can be achieved by deploying more nodes. This is not always true. When more nodes are deployed, more transmissions of packets occur, which requires more energy consumption. Again, increases in the number of nodes for deployment increases the system’s costs.

M. Akter et al. [5] proposed a model for the energy-efficient tracking and localization (EETL) of objects, which focused on enhancing the accuracy of object detection and localization. This model detects and tracks objects at the cluster boundary using energy-efficient incremental clustering based on the Gaussian adaptive resonance theory. The algorithm can learn, create, update and retain clusters incrementally through online learning. Energy efficiency is achieved using online clustering and by retaining past cluster information. For object localization, a trilateration-based approach is used. The drawback of this model is that more communication with the sink occurs, which leads to energy wastage. As the LEACH protocol is used to cluster nodes, the issue of energy balancing is ignored, which may lead to network partitioning. No clear definition of the boundary node is given, which raises the question of the applicability of online cluster creation at the boundary region of the cluster. Again, ART is not applicable when there is a single boundary node in a cluster, as ART uses the competitive learning method. The model does not address network coverage and connectivity. When an object is sensed by a cluster head (CH), it sends information to the sink. The sink activates all of the members of that cluster for object localization purposes. The activation of all of the sensor nodes in a cluster may be unnecessary, as the trilateration method is used for object localization.

Khalid Jamal Jadaa et al. [6] proposed a model for the detection and localization of objects in WSNs using the probabilistic model, which focuses on the issue of the reliability of object sensing information in surveillance systems. According to the authors, the use of a predefined sensing range for sensors for object detection does not reflect sensor reliability, object characteristics or environment conditions. Hence, the detection information cannot be fully relied upon. Thus, the predefined sensing model or binary sensing model can be replaced with the probabilistic sensing model for the accurate detection of the presence of objects in the AOI. The approach focuses on enhancing object detection and localization accuracy. It does not address the lifetime of the network. The concept is meaningless in a network where no attempt is made to lengthen the lifetime of the network.

P. Leela Rani and G. A. Sathish Kumar [7] proposed an energy-efficient object detection and tracking model named the TDTT model. The model uses the concept of cliques to cluster the nodes and a Kalman filter for object localization. It can regulate the number of nodes engaged in object detection and localization at a particular period of time. The authors studied the maximum error in target position estimation based on the node density and sensing range of sensors. The clique formation is carried out each time the object’s position changes. The proposed model does not address network coverage and connectivity, which has a major impact on the tracking results.

Koyuncu, H. and Koyuncu, B. [8] proposed a dynamic object trajectory localization technique based on the Kalman filter and Artificial Neural Network (ANN), which was studied in a closed indoor environment. The model is used to determine the location of a person in a 2D area. The ANN algorithm is used to determine a moving person’s location from a fingerprint map consisting of RSSI information collected from RFID nodes. The Kalman filter is used to model the trajectory of a moving person. The proposed technique focuses on the minimization of errors in the location of an object.

Ammar Hawbani et al. [9] proposed a group-based multi-object location tracking model intended for scalable WSNs and focused on energy-efficient tracking. The nodes deployed are partitioned into a number of groups based on their maximum coverage region, and each group consists of nodes and a leader, which controls the operation inside the group. The proposed algorithm consists of two tiers. The first tier is called the ‘Notification Tree’ and is associated with an activation mechanism, data cleaning mechanism and energy balancing mechanism. The second tier is called the ‘Hierarchical Spanning Tree’ and is associated with the data reporting mechanism and lifetime-enhancing mechanism. GLT reduces the communication overhead and thereby saves energy and provides improved tracking accuracy. However, it ignores the wastage of energy due to the overhead generated due to the bulk amount of node deployment.

Wang, T. et al. [10] studied the problem from the perspective of sensing and decision fusion and proposed a probabilistic object detection model which uses a probabilistic sensing model to sense objects. The model uses spatiotemporal information at the CH to make final decisions about an object’s presence. The authors claimed that their model guarantees a high probability of detection and a low probability of false alarm. The model does not address energy efficiency and object localization accuracy.

Wamuyu, P. K. [11] proposed a conceptual framework for a WSN-based cattle detection and recovery system which focuses on detection accuracy. The movement of cattle is tracked at the village level and in harsh terrain, and their location is estimated using the DV-HOP algorithm in order to recover cattle in the case of theft. The framework does not address effective power management in the network, which greatly affects the lifetime of the network.

Hirpara, K. and Rana, K. [12] proposed an energy-efficient target tracking model in which a trade-off between tracking accuracy and power consumption in tracking is attempted. The model combines the feature of node clustering and prediction-based collaborative tracking for effective tracking. The model uses a mobile BS for data collection from cluster heads and hence decreases the level of redundant data transmission among the cluster heads. The BS predicts the next location of the target using a Kalman filter and sends information regarding the predictions to the CH adjacent to the predicted location, and then three other nodes are awakened, and the target location is calculated using a trilateration algorithm by the leader node selected by the CH. The leader node sends the information regarding location to the CH where this calculated value is compared with the predicted location value by the mobile BS. If the difference between them is greater than the predefined threshold value, the CH sends this difference value to the BS, which updates the predicted value.

Wei C. et al. [13] studied the problem of determination of the location of a target in the AOI. They proposed the use of an error-correcting code with the target localization process to minimize target localization errors. This error-correcting code uses the weighted average sensor positions with binary weightings from local decisions to minimize the error. It uses information regarding sensor locations to determine the location of the target. The authors claimed that the model provides accurate object location information even when some nodes are under a byzantine network.

Calafate, C. T. et al. [14] proposed a comprehensive model for target detection and tracking which combines a tracking algorithm with a routing algorithm. The model consists of two algorithms: an intruder tracking algorithm and the Mobile Sink Routing for Large Grid (MRLG) algorithm. Routing information is also used by the nodes for packet transmission to the sink. The model uses a mobile sink to minimize delays in transmission and packet loss. The mobility of the sink increases the generation of overhead information.

Soderlund, A. A. and Kumar, M. [15] proposed a new node-clustering approach named the Information Guided Rapid Clustering Algorithm (IGRCA) for applications in WSN-based multi-target tracking, which helps to minimize the target localization error and avoids the loss of target tracks. This study involved the optimization of the cluster formation process and the selection of optimum sensors within the cluster for the measurement of object location. A three-step process is applied in order to minimize the object localization error. In the sensing feasibility step, only the sensors that can sense the target’s presence at any time are selected as feasible nodes, and a cluster is created. In the information utility step, feasible nodes that have high potential to reduce the uncertainty in target tracking at a particular time are selected based on the information collected. Then, the optimum node is selected to measure the target’s location. The third step involves reducing the computational cost of routing information from the sensor to the processing node and vice versa.

Cao, X. and Madria, S. [16] proposed an object tracking framework which predicts the trajectory of a moving object using a sequence-to-sequence learning model and only wakes up the sensors that fall within the predicted trajectory to continue the tracking operation. The framework translates the object’s moving trajectory to a sequence of cascaded hyperbolas, encodes these hyperbolas with the DV-Hop algorithm and generates routing constraints. A specially designed control packet containing these constraints is used to prevent nodes that are not in within the predicted trajectory from awakening and participating in the tracking process. The proposed framework maintains location anonymity by only transmitting information regarding hop count relating to the location of object. This framework requires a predefined trajectory for encoding.

Chen, H. W. and Liang, C. K. [17] proposed an energy-efficient algorithm which considers the problem of the coverage of a moving object in a predefined trajectory in a mobile wireless sensor network with a limited number of mobile nodes. Energy efficiency is achieved by regulating the movement of mobile sensors while continuing the coverage of the object’s trajectory at a particular period of time. The paper focuses on the optimization of mobile sensor node paths in order to cover the object’s trajectory using the Genetic Algorithm (GA) and the Discrete Particle Swarm Algorithm (DPSA). It can be used to track single objects and predefined object trajectories.

Barijini et al. [18] proposed an energy-efficient target tracking algorithm called the NGEKF algorithm, in which energy efficiency is achieved through sensor scheduling. An optimal sequence of eight sensor nodes is scheduled to determine the presence of an object and its position. The model is designed for static sensor nodes. The model does not address the achievement of detection accuracy.

Lv C. et al. [19] proposed a prediction-based object tracking model named Measurement-Compensation-based Mixture-Population-Monte-Carlo (MC-MPMC). This model achieves energy efficiency by using predicted locations to track targets. The model also has the ability to compensate for missed predicted locations or false location estimations and thereby avoids the degradation of tracking behavior.

Liu F. et al. [20] proposed a novel Adaptive-Dynamic-Programming-based Multi-Sensor Scheduling (ADP-MSS) algorithm for collaborative target tracking in energy-harvesting WSNs. It schedules multiple sensors for each time step over an infinite horizon to achieve a high level of tracking accuracy.

Qu Z. and Li B. [21] proposed a clustering method named the Tracking-Anchor-based Clustering Method (TACM) to achieve energy efficiency in WSN-based target tracking applications. They introduced tracking anchors which activate nodes depending upon the object position and form clusters. It achieves energy efficiency by minimizing the overhead generated from cluster creation and minimizing the number of transmissions.

Tang Chao et al. [22] proposed an algorithm for object detection and localization in WSNs. They designed a weight-based distribution algorithm which assigns stability weight to each sensor according to their survival prediction. It suppresses signal measurement dropout and automatically adjusts the weight of a sensor based on the measurement of error covariance in order to achieve a high level of tracking accuracy.

Shahbazian, R. and Ghorashi, S.A [23] proposed a distributed cooperative target detection and localization model for decentralized wireless sensor networks. This includes distributed-consensus-based target detection and distributed-consensus-based target localization. This model considers the case of detection in the case of communication link failure. Individual sensor nodes make the decision about a target’s presence in their sensing range, determine the location of the object and exchange this information with their neighbor.

Most researchers have attempted to enhance the application lifetime while achieving target detection accuracy at the cost of high node density. They neither considered the system cost nor the accuracy of object localization. Some researchers focused on the accuracy of object detection while achieving energy efficiency by limiting the detection to the predefined target trajectory. In particular, some researchers only focused on node clustering and node scheduling for energy efficiency.

The features incorporated in our framework and in other previous research studied by us are shown in Table 1. In previous research, energy efficiency is mainly achieved by node clustering, optimizing the mobile node trajectory and sleep scheduling. However, in the current study, additional points are considered. These include node optimization, minimizing the number of transmissions by minimizing the number of nodes and minimizing the number of clusters. Table 2 and Table 3 give a description of the comparative study of the past research described in the literature.

## 3. Proposed Energy-Efficient Object Detection and Tracking Framework

### 3.1. Preliminaries

#### 3.1.1. Assumptions

- AOI is a two-dimensional rectangular area.
- The CH and BS are synchronized.
- The CH and cluster members are synchronized.
- The communication range of each sensor is twice its sensing range.
- The energy consumed by sleeping nodes is neglected.
- The sensing area of each sensor is circular.
- The binary sensing model is used.
- When the network coverage is high, the network connectivity is high.

#### 3.1.2. Network Model

**Figure 1:**
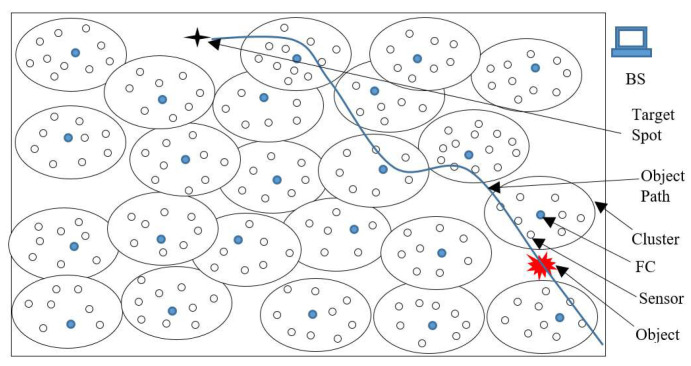
Network model.

Consider a heterogeneous wireless sensor network consisting of both static and mobile nodes. All of the nodes are divided into a number of clusters, as shown in Figure 1. Mobile nodes are used to enhance the network coverage. After deployment, no nodes change their position throughout the operation. An object is planned to move randomly with uniform velocity.

The AOI is obstacle free. Initially, all of the sensor nodes are in the sleep state. When a sensor node senses the presence of an object, it sends sensing information to its CH. The CH acts as a Fusion Centre (FC), where all of the local decisions are fused and a final decision about the object’s presence is made. The CH sends this decision information to the BS. If the decision is positive, the BS determines the object position and predicts the next location of the object. Based upon the predicted information, the BS sends an alert message to the nearest CH. If the predicted location is not within the sensing area of any cluster, then the BS declares the last node that sensed the object as the CH, a new on-demand cluster is created and tracking continues. This increases the tracking accuracy.

#### 3.1.3. System Architecture

**Figure 2:**
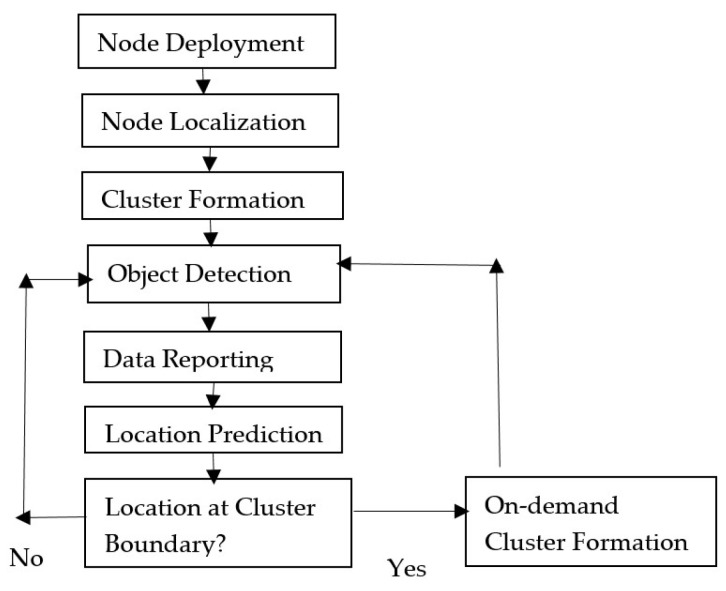
System architecture of EEODTF.

**Figure 3:**
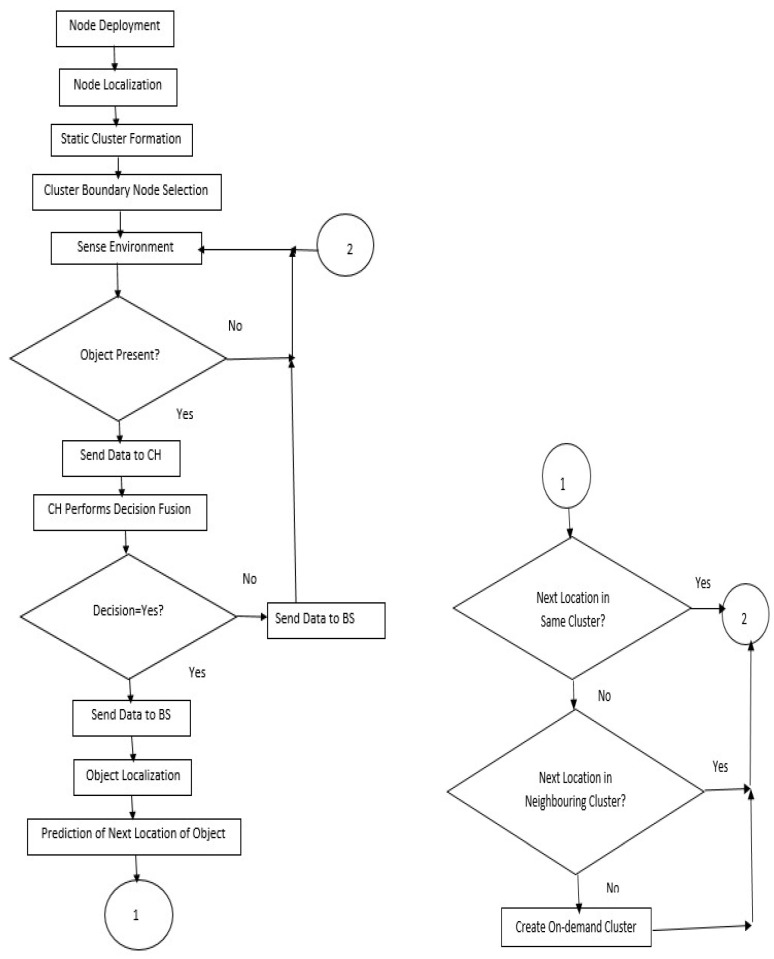
System flow chart of EEODTF.

Figure 2 shows the system architecture. The system flow chart is given in Figure 3. The object emits a signal, and this signal is sensed by sensors. The sensors measure the distance between the object and the sensor and then calculate the signal strength.

If the signal strength is greater than the threshold, then the object is present. Otherwise, the object is absent. The sensor sends this information to the CH. The CH aggregates this information from the sensor nodes, and a final decision is made on the basis of the aggregated signal. The final decision is reported to the BS. Then, the BS initiates the process of object localization.

#### 3.1.4. Prediction Model

This framework uses a linear-prediction-based mechanism to track objects. In this case, the algorithm predicts the next location of the object in the AOI, and based on the location information, specific nodes are activated to continue the tracking. The remaining nodes are kept in the sleep state. The next location of the object is calculated based on the current and previous location. Let *(x_i_,y_i_)* and *(x_i−_*_1_*,y_i−_*_1_*)* be the current and previous positions of the object in time *t_i_* and *t_i-1_*, respectively. The object speed *v* is calculated using the following Equation (1):

(1) $$v=\frac{\sqrt{{\left(x_{i}-x_{i-1}\right)}^{2}+{\left(y_{i}-y_{i-1}\right)}^{2}}}{t_{i}-t_{i-1}}$$

The direction of the object is determined using Equation (2):

(2) $$\theta =\cos^{-1}\frac{x_{i}-x_{i-1}}{\sqrt{{\left(x_{i}-x_{i-1}\right)}^{2}+{\left(y_{i}-y_{i-1}\right)}^{2}}}$$

The next location of the object *(x_i+_*_1_*,y_i+_*_1_*)* is calculated using Equation (3):

(3) $$x_{i+1}=x_{i}+vt\;cos\;\theta \;y_{i+1}=y_{i}+vt\;sin\;\theta$$

#### 3.1.5. Energy Model

If the packet size is *k*-bit, then the energy consumption at the sender’s end is given in the following Equation (4):

(4) $$E_{TX}\left(k,d\right)=\{\begin{array}{c} k*\;E_{elec}+k*E_{amp}*\;d^{2},\;if\;d<d_{0}\\ K*\;E_{elec}+K*\;E_{fs}*d^{4},if\;d\geq \;d_{0} \end{array}$$

where $E_{TX}$ is the energy required for packet transmission, *d* is the distance between the source and destination and $d_{0}$ is the threshold distance.$\;E_{elec}$ is the base energy required to run the transmitter or receiver.$\;E_{fs}$ and $E_{amp}$ are the unit energy required for the transmitter and amplifier.

(5) $$d_{0}=\sqrt{\frac{E_{fs}}{E_{amp}}}$$

The energy $E_{RX}$ required to receive *k*-bit messages is given in Equation (6):

(6) $$E_{RX}(k)=k^{*}E_{elec}$$

#### 3.1.6. Problem Statement

Three major issues in WSN-based object detection and tracking are energy efficiency, detection accuracy and object localization accuracy. From the related literature studied, we did not find a complete study in which it was attempted to minimize the energy consumption in different stages of an object tracking application and ensure high levels of object detection and localization. Some studies are based on object localization accuracy and energy efficiency in a network. Very few studies are based on node deployment and node localization with the scheme of target tracking. No studies mention energy-efficient node deployment which guarantees the maximum coverage of the AOI and energy-efficient routing for the minimization of data loss. Similarly, none of the literature studied focuses on improving the object localization accuracy by improving the node localization accuracy. No studies discuss the importance of the adaptive threshold required for object detection, as the distances between the cluster head and member nodes are not the same in all cases. In this study, we did not consider the adaptive threshold. The research problem is defined as follows:

Given *n* number of static nodes and *m* number of mobile nodes for the operation of object detection and tracking, design a complete framework that minimizes energy consumption for a tracking operation while maximizing the object detection and localization accuracy.

### 3.2. Proposed EEODTF Framework

#### 3.2.1. Overview of the Proposed Framework

**Figure 4:**
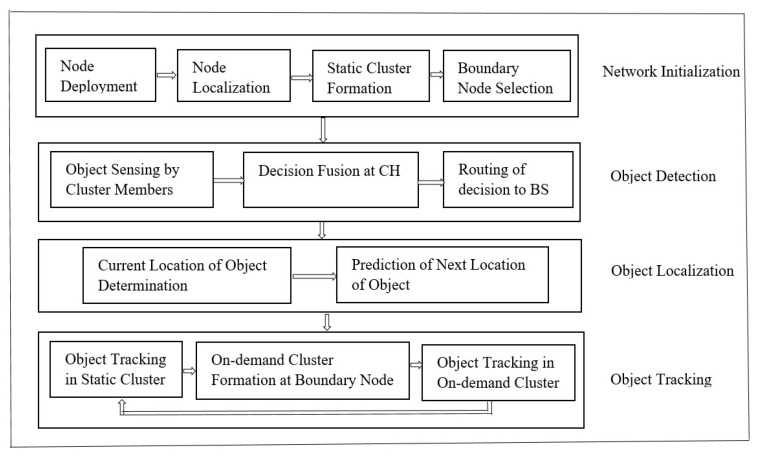
Proposed energy-efficient object detection and tracking framework.

The fundamental problem addressed in this research is energy-efficient tracking which does not compromise object detection accuracy and object localization accuracy. Figure 4 outlines the overview of the proposed framework. The framework consists of four major parts: network initialization, object detection, object localization and object tracking.

##### Network Initialization

Heterogeneous nodes are randomly deployed in a two-dimensional area of interest. A mixture of static nodes and mobile nodes are used for deployment. Two types of mobile nodes are used: ordinary mobile nodes and powerful mobile nodes. The number of nodes deployed is optimized using the PSO algorithm. Initially, the nodes are deployed randomly. Then, to cover up the coverage hole in the network, the locations of the mobile nodes are adjusted. This maximizes the network coverage and connectivity while controlling the cost of the network. The network is considered as a grid for the optimization of the trajectory of mobile nodes. The movement of low-powered mobile nodes is optimized using an eight-neighborhood algorithm, whereas the movement of high-powered mobile nodes is optimized using the PSO algorithm [24]. After the deployment task, the mobile nodes do not move any further. Then, the BS divides the nodes into different clusters. The cluster formation process is adaptive and energy efficient. Then, the boundary nodes of each cluster are determined using the technique used in [5]. The nodes are localized using the range-free localization method by BS. The Hybrid Particle Swarm Optimization–Grey Wolf Optimization algorithm with the poor-for-change strategy is used for node localization.

##### Object Detection

After the initialization of the network, the BS initiates object detection. It instructs the cluster head (CH) to inform it about the presence of an object in their area, and in turn the CH passes this information to its cluster members. The CH runs a scheduling algorithm which determines the time taken for each member to communicate with the CH. When a member node senses the presence of an object in its sensing area, it passes the information regarding its decision to the CH. The CH collects decision-related information about an object’s presence from its other members, fuses all of the decisions and sends this information to the BS. The BS issues a notification to the nodes nearest to the object’s position via their CH to wake up and help in the process of localization and tracking. The remaining nodes remain in the sleep state at this time. The majority voting technique is used to make the final decision.

##### Object Localization

After the confirmation of the object’s presence in the cluster area, the object localization process is initiated by the BS. The three nodes nearest to the object’s current position are selected by the BS, and the trilateration algorithm [5] is applied to determine the location of the object in each step. The next location of the object is predicted using the past location information, the velocity of the object and time-related information.

##### Object Tracking

The object is continuously tracked by the CH, and object-presence-related information is sent to the BS as long as the object is in the cluster area. When the object is at the boundary area of the cluster, the CH shares the object’s current location, predicts the next location of the object with the neighboring CH and waits for a reply from them. CHs present at a one-hop distance are neighbors of a CH. If the next predicted location is in any of the neighboring cluster areas, then that CH sends a positive reply to the sender CH. Otherwise, it sends a negative reply to the sender CH. In case of a positive reply, the sender CH transfers the control of object tracking to the neighboring CH. When the CH receives negative replies from all of the neighboring clusters, the BS selects a suitable boundary node to act as a CH for on-demand cluster creation. The BS allows the formation of an on-demand cluster with the said CH, and the nearby nodes join to this cluster as members. The BS shares on-demand cluster-formation-related information with the CHs. Then, the control of object tracking is transferred to the CH of the on-demand cluster and tracking continues. The CH of the on-demand cluster continues tracking until the object is in its area.

#### 3.2.2. Proposed Energy-Efficient Object Detection and Tracking Algorithm

Algorithm 1 describes the complete EEODTF algorithm for moving object detection and tracking in WSNs. This algorithm is an integration of different steps in the tracking process such as node deployment, node localization, cluster formation, object detection, object localization and the routing of tracking information to BS. In each step, the energy consumption is minimized where possible. Additionally, the detection accuracy is maximized, and the object localization error is minimized where possible.
**Algorithm 1**: Energy-efficient object detection and tracking.***Requires****: Network Initialization, Cluster Formation, Object Trajectory Pattern, Time of simulation* ***Ensures****: Energy-efficient robust object tracking and localization* *1.* *Initialize the network**2.* *Deploy the nodes using **Algorithm 2.****3.* *Localize the sensor nodes and form static cluster using **Algorithm 3**.**4.* *For t=1:time_of_simulation** I. Create Cluster using **Algorithm 4.*** * II. Create cluster boundary and determine the boundary nodes of each cluster using **Algorithm 5*** * III. If presence of object is sensed by a static cluster node*   *a.* *Perform Decision Fusion at CH for final decision using **Algorithm 6***  *b.* *Report decision to BS using **Algorithm 7***  *c.* *If decision is positive**   i. BS awakes necessary nodes for object localization* *   ii. Location of object is estimated using **Algorithm 8*** *   iii. Next location of object is predicted using Equation (3)* *   iv. If next location of object is outside currently tracking cluster area* *    If the next location of object is in the none of the neighboring cluster area*     *(a)* *Select boundary node as CH*    *(b)* *Create On-demand cluster and continue tracking using **Algorithm 9****    Else* *       Continue tracking in neighboring cluster* *              End* *            Else* *              Continue tracking in same cluster* *            End* *           Else* *            Nodes go to sleep mode* *           End* *          Else* *           Nodes go to sleep mode* *          End* * End*

**Figure 5:**
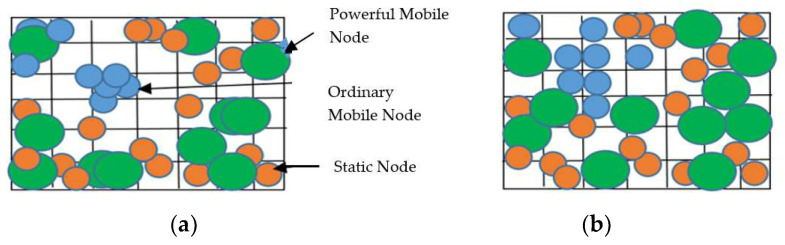
(**a**) Before network coverage optimization (**b**) After network coverage optimization.

Algorithm 2 describes the energy-efficient node deployment, node localization and static cluster formation. It is sensible to have maximum area coverage with the minimum number of nodes in terms of the network cost and energy efficiency. The AOI is divided into a number of cells of equal size, and each cell has eight neighboring cells. A hole is a cell in the network which is not in the sensing range of any sensor, either fully or partially. Three types of nodes are used for deployment: static nodes, ordinary mobile nodes and powerful mobile nodes. An ordinary mobile node, the majority of the sensing area of which is overlapped by other sensors, is relocated to any of the immediate neighboring cells if it is a hole. This is known as the eight-neighborhood technique. Distant holes are filled by moving eligible powerful mobile nodes to these locations using the PSO algorithm. Both the eight-neighborhood technique and the PSO algorithm are used to optimize the trajectory of mobile nodes so that less energy will be consumed while maximizing the network coverage; see Figure 5a,b. The objective function used for the optimization of the number of nodes (both static and mobile) when using PSO is given in Equation (7).

(7) $$\begin{array}{l} Minimize\;f\left(n,m,p\right)=n+m+p\\ \mathrm{Subject}\;\mathrm{to}\;A_{cov}\geq 0.99\times Area_{AOI}\;\;\;,\;n>0\;,\;m>0,\;p>0 \end{array}$$

where $A_{cov}=\sum_{i=1}^{n}\pi r_{1}^{2}+\sum_{j=1}^{m}\pi r_{2}^{2}+\sum_{k=1}^{p}\pi r_{3}^{2}\;$, where *n, m, p* are the number of static nodes, the number of ordinary mobile nodes and the number of powerful mobile nodes, respectively, and *r_1_,r_2_,r_3_* are their sensing ranges.$\;A_{cov}$ and $Area_{ROI}$ are the coverage area and the area of the AOI, respectively.

(8) $$\begin{array}{l} Actual_{cov}=A_{cov}-A_{ov} \end{array}$$

where $Actual_{cov}$ and $A_{ov}$ are the actual coverage area and overlapping area, respectively.

The objective function used for the optimization of the trajectory of mobile nodes when using PSO is given in Equation (9)*. (x_old_,y_old_)* is the old position of the mobile node. *(x_new_,y_new_)* is the new position of the mobile node, and *(xm×ym)* defines the AOI size. *U_u1_* and *U_u2_* define the required updates to the position.

(9) $$\begin{array}{l} Minimize\;A_{ov}\\ \mathrm{Subject}\;\mathrm{to}\;f\left(x_{new},y_{new}\right)=\left(\left(x_{old}+U_{u1}\right),\left(y_{old}+U_{u2}\right)\right) \end{array}$$

where $0<U_{u1}<xm$ and $0<U_{u2}<ym$
**Algorithm 2**: Energy-efficient node deployment*1.* *Initialize network parameters**2.* *Determine no. of static nodes, ordinary mobile nodes and powerful mobile nodes required for desired coverage in AOI using Equation (7) and deploy them randomly**3.* *Divide AOI into grids and define area of each rectangular cell**4.* *Determine the hole area in each cell and calculate its size**5.* *Determine the hole segments**6.* *//Fill the holes with the help of mobile nodes**7.* *Determine the eligible mobile nodes for hole filling**8.* *If eligible_node_type == ordinary mobile node**  i. Move the ordinary mobile node to the hole segment in adjacent cells of the cell where currently the node is present using 8-neighborhood technique.* *  ii. Update the ordinary mobile node location* *   Else if eligible_node_type == powerful mobile node* *  iii. Move the powerful mobile node to cover a large hole segment in the network using PSO algorithm in order to fill it. Use Equation (9)* *  iv. Update the powerful mobile node location* *  End* *9.* *Repeat steps 8 and 9 until desired area coverage is achieved*

The node localization method greatly affects the object localization performance when the range-free method is used for object localization. When the node localization error is lower, the object localization error is lower. DV-Hop [25,26] is a commonly used node localization algorithm, but a limitation is that it causes more node localization errors. PSO and Grey Wolf Optimization (GWO) [27] are two nature-inspired optimization algorithms. Algorithm 3 uses the hybrid DV-Hop-PSO-GWO algorithm with the poor-for-change strategy for node localization. We tried to minimize the node localization error with the hybrid PSO-GWO algorithm with the poor-for-change strategy. According to this strategy, the poor individuals in the population are modified to increase their strength.

Let *{(x*_1_*,y*_1_*),(x*_2_*,y*_2_*),(x*_3_*,y*_3_*),…,(x_n_,y_n_)}* be beacon node positions and *{d*_1_*,d*_2_*,d*_3_*,….,d_n_}* be the distances between the unknown nodes and each of the beacon nodes. Let *(x_a_, y_a_)* be the actual location of the unknown node. Let *{e*_1_*, e*_2_*, e*_3_*,…,e_n_}* be the differences between the estimated distance and the actual distance between each of the beacon nodes and unknown nodes. DV-Hop uses the following set of equations to calculate node location (see Equation (10)):

(10) $$\{\begin{array}{c} \sqrt{{\left(x_{a}-x_{1}\right)}^{2}+{\left(y_{a}-y_{1}\right)}^{2}}=d_{1}+e_{1}\\ \sqrt{{\left(x_{a}-x_{2}\right)}^{2}+{\left(y_{a}-y_{2}\right)}^{2}}=d_{2}+e_{2}\\ \ldots \\ \sqrt{{\left(x_{a}-x_{n}\right)}^{2}+{\left(y_{a}-y_{n}\right)}^{2}}=d_{1}+e_{1} \end{array}$$

Equation (10) can be rewritten as in Equation (11).

(11) $$e_{i}=|\sqrt{{\left(x_{i}-x_{a}\right)}^{2}+{\left(y_{i}-y_{a}\right)}^{2}}-d_{i}|\;,\;for\;i\;=\;1,2,...,n$$

The fitness function used for node localization is given in Equation (12).

(12) $$Minimize\;f\left(e\right)=\sum_{i=1}^{n}{\left(\frac{e_{i}}{hop_{i}}\right)}^{2}$$

where *hop_i_* is the minimum hop count in between the unknown node and beacon node *i*, and *e* is the localization error for the unknown node.

**Algorithm 3**: Hybrid DV-Hop-PSO-GWO algorithm for node localization.
*1.* 
*Deploy ‘n’ beacon nodes and ‘m’ unknown nodes*
*2.* 
*Beacon nodes determine their own location*
*3.* 
*Calculate the distance between each pair of beacon node and broadcast this information along with location information*
*4.* 
*For i=1 to m*

*    E_i_=0*

*    For j=1 to n*

*    a. Calculate minimum hop_j_ for each i from each j*

*    b. Calculate distance between each i from each j*

*    c.Calculate the error e_j_ between actual distance and calculated distance for each j using equation (10)*

*    d. E_i_=E_i_+ (e_j_ / hop_j_)*

*    End*

*   End*

*5.* 
*Calculate the average localization error* $\frac{\sum_{i=1}^{m}E_{i}}{m}$

*6.* 
*Minimize the average localization error*

*    i. Initialize the population and all parameters*

*    ii. Evaluate fitness of each individual in the population using Equation (12)*

*    iii. Find alpha, beta, delta, pbest, gbest*

*    iv. Find X_α_, X_β_, X_δ_*

*    v. Update the current individual according to the poor-for-change strategy*

*    vi. Check for boundary condition violation*

*    vii. Update the velocity and position all individuals*

*    viii. Sort the population according to the decreasing order of fitness of individual*

*    ix. Update pbest, gbest, alpha, beta, delta*

*    x. Update GWO parameters*

*    xi. Repeat steps iv to x until termination criteria is reached*

*7.* 
*Output the best result*



Algorithm 4 deals with the creation of an energy-efficient cluster. The PSO algorithm is used for cluster head (CH) selection, and Euclidean-distance-based clusters are created. Let *S={s_1_,s_2_,…,s_n_}* be the set of sensor nodes, *RE={RE_1_,RE_2_,…,RE_n_}* be the current energy level of the nodes, *ND = {ND_1_,ND_2_, …., ND_n_}* be the node degree of each sensor node and *ED = {ED_1_,ED_2_,…,ED_n_}* be the set of Euclidean distances between each node and the BS. The number of CH is represented as *k* for a given area and is randomly taken. The fitness function for the selection of a node as a CH is given in Equation (13).

(13) $$fit_{i}=m\times \left(RE_{i}+ND_{i}\right)+\left(1-m\right)\times \left(\frac{1}{ED_{i}}\right)$$

where *RE_i_* is the residual energy of node *i*, *ND_i_* is the node degree of node *i* and *ED_i_* is the Euclidean distance between node *i* and BS and *m*∈*[0, 1]*.

Let *X_i_={x_1_,x_2_,x_3_,...,x_n_}* represent the *ith* solution in the population. The fitness of the solution is calculated using Equation (14).

(14) $$F\left(X_{i}\right)=\sum_{i=1}^{k}fit_{i}$$

**Algorithm 4**: PSO Euclidean-distance-based cluster formation
*1.* 
*Initialize the network parameters, PSO algorithm parameters and initialize the population.*
*2.* 
*For i= 1 to no_of_iteration*

*  //Select the CHs*

*  (a)* 
*For count=1 to no_of_rounds*

*  (i)* 
*For i=1 to no_of_rows in population //Calculate fitness*

*    Calculate the fitness of each search agent using Equation (13)*

*   End*

*  (ii)* 
*Determine the fittest solution comprising of CHs using Equation (14)*
*  (iii)* 
*Find particle best P_best_ and global best G_best_*
*  (iv)* 
*Update velocity and position of particles using following equations and generate the new solution*

*    a)* 
v_i_(t + 1) = w*v_i_(t) + c_1_r_1_[ Pbest_i_ (t) − x_i_(t)] + c_2_r_2_[Gbest(t) − x_i_(t)]
*    b)* 
*x_i_(t + 1) = x_i_(t) + v_i_(t + 1)*

*End*

*(c) Form cluster on the basis of proximity using Euclidean distance formula*


The cluster boundary, which is roughly a circle, is determined according to the communication range of the CH. This is because two sensors can only communicate with each other when they are in each other’s communication range. In cluster-based network architecture, a member of one cluster cannot directly communicate with a member of another cluster, even if they are in each other’s communication range. This is a drawback of cluster-based continuous object tracking in WSNs. If the next location of the object is outside the cluster boundary and this location is not in any of the cluster areas, then a false negative will occur. To bridge this gap, we used Algorithm 5, which determines the boundary node for each cluster to facilitate the creation of an on-demand cluster to manage the situation of a false negative or a target being missed.
**Algorithm 5**: Boundary node selection algorithm.*1.* *For i=1:number_of_clusters** i Determine cluster boundary considering the sensing range of cluster members* * ii Determine the nodes adjacent to boundary* *  End* *2.* *For i=1:number_of_clusters**    Member(i)=member nodes of cluster i* *    Size(i)=size of cluster* *    For j=1:Size(i)* *3.* *For i=1:number_of_clusters**    Member(i)=member nodes of cluster i* *    Size(i)=size of cluster* *    For j=1:Size(i)* *      If a node belonging to Member(i) has overlapping communication range with the node of adjacent cluster then that node is declared as boundary node* *    End* *End*

An object enters the AOI. The node activation schedule run at the BS determines which nodes will remain in the active state and which will be in the sleep state in an instant. Active sensors periodically emit radio signals and measure reflected signals in order to detect the presence of an object. In the absence of an object, the measured signal strength is negligible, whereas in the case of the presence of an object, it is comparatively strong. Again, the received signal strength is high if the node position is closer to the object’s position, and it is weak in the case of a distant sensor. Object detection operation is initiated and regulated by the BS. Let the sensor *i* be *d_i_* meters away from the object. Then, the attenuated signal energy *sx_i_* is given in Equation (15).

(15) sxi={E0(dir)k ,  if di>d0E0 ,         if di≤d0

where *E_0_* is the original signal strength, *r* is the size of the object, *k* is the attenuation factor which ranges from 2 to 5 and $d_{0}$ is the reference distance.

Again, the received signal is not free from environmental noise. Let *H*_0_ represent the object’s absence and *H*_1_ represent the object’s presence. Sensors detect the presence of the object independently. Then, we can write Equation (16) as follows:

(16) $$H_{0}:y_{i}=\eta_{i}\;\;\;\;\;\;\;\;\;\;\;and\;\;\;\;\;H_{1}:y_{i}=sx_{i}+\eta_{i}$$

where *y_i_* is the received signal strength and *η_i_* is random white Gaussian noise with zero *µ_i_* and variance of *σ_i_^2^*. The decision regarding a signal’s presence (denoted as 1) or absence (denoted as 0) is modelled as in Equation (17).

(17) $$u_{i}=\{\begin{array}{l} 1,\;\;\;\;if\;y_{i}>\theta \\ 0,\;\;\;if\;y_{i}\leq \;\theta \end{array}$$

where *u_i_* is the decision at sensor *i* and *θ* is the predefined threshold.

In our work, we tried to calculate the relative received signal strength when an object is detected based on the reference distance and reference signal strength. Noise variance and attenuation are kept constant for all of the nodes. Let the reference signal strength be 20 dB and the reference distance be 10m. If the object is 20 m away from the node, then the received signal strength is 40 dB.

When a sensor senses the object’s presence, it reports to its CH. Each sensor takes three measurements when it senses the object. If any two measurements are positive, then the sensor reports a 1 to CH. This will avoid a false alarm. The CH collects information from all the members and makes a final decision based on the collected information using the majority rule. According to this rule, if the majority of the members signal 1, then the final decision is 1. Otherwise, the final decision is 0. The final decision of the CH is reported to the BS. Algorithm 6 describes the decision fusion algorithm.

**Algorithm 6**: Decision fusion algorithm.
*1.* 
*Num1 = 0;*
*2.* 
*For i = 1:no_of_members_in_cluster*

* i. Num = 0;*

* ii. For count=1:3*

*   a. Measure attenuated signal energy sx_i_ using Equation (15)*

*   b. Calculate the received signal strength y_i_ using Equation (16)*

*   c. Calculate u_i_ is Equation (17)*

*   d. If u_i_ ==1*

*      Num=Num+1*

*     End*

*    End*

* iii. If Num>1*

*      lu_i_=1*

*    Else*

*      lu_i_==0*

*    End*

* iv. Report ui to CH*

* v. If lui==1*

*      Num1=Num1+1;*

*    End*

*  End*

*3.* 
*//Decision Fusion at CH*

*  If Num1 > (0.5× no_of_members_in_cluster)*

*    Decision=1*

*   Else*

*    Decision=0*

*   End*

*4.* 
*Report Decision to BS*



An energy-efficient path is used for communication between the CH and BS. By requiring an energy-efficient path, the objective is to balance the energy throughout the network and minimize the data loss due to node failure which is often caused by a shortage of energy. For route selection, a graph is constructed with nodes which are the CHs and BS. The edges of the graph are the connectivity in the network. The member nodes of two different clusters cannot communicate with each other, and hence there should not be any edge between them. An edge exists in between the CH and a member node. The edge cost is calculated using Equation (18) considering the residual energy of the node, the node degree and the distance between the node and the BS.

(18) $$w_{ij}=\frac{E_{f}\times d_{ij}}{E_{j}}\times \frac{E_{i}}{E_{j}}+\frac{ND_{j}}{E_{j}}$$

where *w(i,j)* is the cost of the edge-connecting source node *i* and destination node *j*. *E _j_* is the residual energy of destination *j*. *E_i_* is the residual energy of source *i*. *ND_j_* is the node degree of node *j. E_f_* is the energy consumed to send a packet to node *j*, and *d_ij_* is the distance between source *i* and destination *j*. The potential of a node *j* to be part of the route is calculated using Equation (19).

(19) $$potential_{j}=w_{ij}\times priority_{j}$$

where *priority_j_* is a random integer number, the value of which lies in the range of 2 to 100. This is used so as not to overburden a particular node with routing and to balance the network energy.

Algorithm 7 describes the energy-efficient routing. This algorithm uses the Firefly algorithm [28] for the determination of energy-efficient paths between the source nodes and BSs. This algorithm is a meta-heuristic optimization algorithm which is based on the flying behavior of fireflies. The objective function of the optimum route selection problem is given in Equation (20).

(20) $$Minimize\;f\left(Cost\left(path\left(i,j\right)\right)\right)=Cost\left(i,u_{1}\right)+\sum_{\left(i,j\right)\in E}^{n_{1}}Cost\left(u_{i},\;u_{j}\right)+Cost\left(u_{n1},j\right)$$

where *n_1_* is the number of intermediary vertices present between *i* and *j* and

(21) $$Cost\left(u_{i},u_{j}\right)=potential\left(u_{j}\right)$$

Fireflies can communicate with each other, search for prey and attract each other using bioluminescence with varied flashing patterns. The brightness of a firefly at position *x* is determined by the objective function. The attractiveness *β* over the *d_ij_* is defined using Equation (22).

(22) $$\beta \left(d_{ij}\right)=\beta_{0}e^{-\gamma d_{ij\;}^{2}}$$

where $\beta_{0}$ is the attractiveness at *d_ij_* = 0. γ is the degree of variation in the attractiveness, and it plays an important role in the speed of the convergence in firefly behavior. The distance between two fireflies *i* and *j* at position *x_i_* and *x_j_* is calculated using Equation (23).

(23) $$d_{ij}=\sum_{k=1}^{d}\sqrt{{\left(x_{i,k}-x_{j,k}\right)}^{2}+{\left(y_{i,k}-y_{j,k}\right)}^{2}}$$

where *k* is the *k*th component of both fireflies.

Equation (24) is used to update the position of the firefly.

(24) $$x_{i}=x_{i}+\beta_{0}e^{-\gamma d_{ij\;}^{2}}\left(x_{j}-x_{i}\right)+\alpha \;S_{k}\left(rand-0.5\right)$$

where α ∈ [0,1] and *S_k_* is the scaling factor for *k = 1, 2, ….. D*, where *D* is the dimension of the problem.

**Algorithm 7**: Energy-efficient path determination.
*1.* 
*S=sourceNode*
*2.* 
*u=S*
*3.* 
*D=destinationNode*
*4.* 
*Create an initial population of fireflies*
*5.* 
*Evaluate the fitness of fireflies using Equation (20)*
*6.* 
*Determine the initial best solution, i.e., the initial best PATH*
*7.* 
*For j=1 to maxIteration*

*  a). For each firefly in the population*

*    PATH={S}*

*    Repeat until firefly k moves to the destination D*

*   i. Determine Adjacent(u) and find the strong neighbor v of u using Equation (21)*

*   ii. Select v as the next node*

*   iii. PATH=PATH U {v}*

*   iv. u= v*

*   v. Update Firefly algorithm parameters using Equation (24)*

*    End*

*   End*

*  b). Find best solution and output corresponding PATH*

*  c). Evaluate the fitness of fireflies using Equation (20)*

*  d). Update best solution*

* End*

*8.* 
*Output PATH*



After receiving information from the CH, the BS initiates the object localization process in coordination with the CH. The trilateration algorithm [5,29] is used for object localization. Algorithm 8 is used for object localization. The three nodes nearest to the object are selected by the BS for the determination of the location of the object. These three nodes act as beacon nodes with known locations. Let *(X*_1_*,Y*_1_*), (X*_2_*,Y*_2_*)* and *(X*_3_*,Y*_3_*)* be the locations of these three nodes. *(X_n_,Y_n_)* is the blind location of the object to be calculated. *D_i_* is the distance between the blind location and sensor node *i*. The sensing range of the node is a circle. Then, the intersection point of these three circles is the location of the object. Algorithm 8 shows trilateration-based object localization.

**Algorithm 8**: Trilateration-algorithm-based object localization.*1.* *Select three nodes of a cluster that sense the object location to act as beacon node**2.* *//Let (X_1_,Y_1_), (X_2_,Y_2_), (X_3_,Y_3_) are three beacon node location and (X_n_,Y_n_) is blind location of object**3.* *For i = 1 to 3*$\;\;\;D_{i}=\sqrt{{\left(X_{i}-X_{n}\right)}^{2}+{\left(Y_{i}-Y_{n}\right)}^{2}}$ *//Euclidean distance between (X_i_,Y_i_) and (X_n_,Y_n_)* *  End* *4.* *//Find (X_i_,Y_i_) and (X_n_,Y_n_)*$\;\;\;X_{n}=\frac{D_{1}^{2}-D_{2}^{2}+X_{2}^{2}}{2X_{2}}$ $\;\;\;Y_{n}=\frac{D_{1}^{2}-D_{3}^{2}+X_{3}^{2}+Y_{3}^{2}-2X_{n}X_{3}}{2Y_{3}}$

After the determination of the location of the object, the next location of the object is predicted using Equation (3). Based on the predicted location, an alert message is sent to the nearest nodes, and they are awakened. If the next location is not in any of the cluster boundaries, then the current boundary node of the current cluster is declared as the CH, and the creation of an on-demand cluster is allowed. Nearby nodes join this CH as its cluster members. The on-demand cluster remains in operation until the next location of the currently moving object is within its cluster boundary. After that, the related information is deleted from the memory of the BS. The objective behind the creation of the on-demand cluster is to increase the object detection accuracy and to minimize the rate of objects going missing. This helps to improve the object localization accuracy if the object is sensed by two or more boundary nodes belonging to different clusters. Let the boundary nodes of a cluster currently tracking the object be *{b*_1_*, b*_2_*,…,b_n_}* and *(x_pred_, y_pred_)* be the predicted next location of the object. Then, the boundary node *b_i_* is selected as the CH of the on-demand cluster, which satisfies the following criteria:

- The current energy level of *b_i_* is greater than the threshold energy level *Th*. All of the *b_i_* that satisfy the energy criteria form *S1*. This is required to avoid the death of the CH during operation.

$$S1=\left\{bb_{1},bb_{2},\ldots ..,bb_{n}\right\}$$

where $RE_{bb}{}_{i}>Th$ and *bb_i_* is the candidate node, satisfying the energy criteria.

There is at least one node which belongs to a different cluster and joins as a member of the cluster whose CH will be *bb_i_*. Let *S2*$=\left\{a_{1},a_{2},\ldots \ldots ,a_{n}\right\}$

be the set of nodes satisfying this criterion.

- The Euclidean distance between *a_i_* and *(x_pred_, y_pred_)* is minimum among all of the candidate nodes, which is calculated using Equation (25).

(25) $$D_{i}=\sqrt{{\left(x_{pred}-a_{x}{}_{i}\right)}^{2}+{\left(y_{pred}-a_{y}{}_{i}\right)}^{2}}$$

Algorithm 9 describes the creation of an on-demand cluster and object tracking within it.

**Algorithm 9**: On-demand cluster creation for tracking.
*1.* 
*For each predicted next location (X_pred_, Y_pred_) of object which is outside current cluster boundary*

*i. CH Sends the predicted location to the neighboring CH*

*ii. If the predicted next location is in any of the neighboring cluster area*

*    a. Neighboring CH sends a response*

*    b. Sender CH transfers the control of tracking to neighboring CH*

*   End*

*iii. If the sender CH gets no reply from any of the neighboring CH*

*    a. Sender CH sends this information to BS and waits for reply*

*    b. BS selects suitable boundary node of that cluster using Equation (25) and declares it as CH of on-demand cluster to be created*

*    c. BS allows its neighboring nodes to communicate with it temporarily for cluster formation*

*    d. CH of on-demand cluster communicates with neighboring nodes and form cluster*

*    e. Sender CH transfers the control of tracking to on-demand CH*

*    f. Use Algorithm 6 for object detection*

*    g. Use Algorithm 8 for object localization*

*   End*


## 4. Result and Discussion

### 4.1. Simulation Environment

The system setup for the simulation of the proposed framework is as follows: a laptop with 16GB RAM, x64-based processor, a 64-bit operating system, a Windows 10 operating system and Matlab R2020b. The object tracking data were stored in the BS, which is equipped with memory devices. The proposed framework consists of four phases: network initialization, object detection, object localization and object trajectory. We performed the simulation-based study of each phase in object detection and tracking. We focused our study on energy-efficient detection and tracking without compromising object detection accuracy or object localization accuracy. The node parameters used in this study (for AOI size 500 × 500 m^2^) are shown in Table 4.

A total of 254 nodes were used in each case, and if the nodes did not overlap, the area coverage remained the same. The BS location was (500,500) m. For convenience, the time taken in the simulation was equal to the number of steps in the object trajectory. Table 5 shows the energy model parameters used in the network. A simulated object trajectory was used in this study. Table 6 shows the algorithmic parameters used for the node deployment algorithm, and Table 7 shows the algorithmic parameters used for the node localization algorithm. Table 8 shows the parameters used to model the object trajectory and object detection. Table 9 shows the parameters used for energy-efficient path selection to report data to the BS.

### 4.2. Simulation Result

#### 4.2.1. Tracking Sequence Analysis

**Figure 6:**
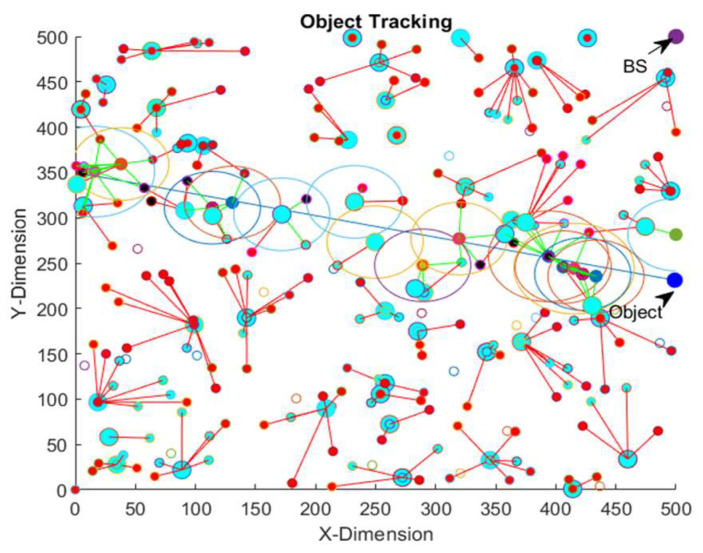
Object tracking in WSN visualization for EEODTF and object trajectory 1.

Figure 6 shows the object’s movement path along with the clusters and on-demand clusters created for the EEODTF framework. The static clusters formed are shown as star structures, and on-demand clusters are shown as circles. The object was shown to move in a straight line. The blue straight line is the object trajectory. The blue-filled circle is the object in the last iteration. The cyan-highlighted CHs are the CHs which took part in the object detection and tracking process.

In the first round of the tracking process, the object was detected by node 30 and node 207, which belonged to cluster 48 and cluster 9, respectively. At that time, the object position was (0,350). Node 30 was a static node, whereas node 207 was a powerful mobile node. Both the sensors sent their measured signals to their respective CHs (node number 23 and 6, respectively). These two clusters were static clusters. Both the CHs carried out their designated tasks. They collected sensing information from their respective members. The members of the cluster with node 23 as the CH were: 2, 23, 37, 32, 154, 158 and 236. The members of the cluster with node 6 as the CH were: 133, 6, 43, 48, 54, 160, 167, 226 and 232. The neighbor of cluster 48 was cluster 50. The neighbor of cluster 9 was cluster 19. The fusion decision of cluster 48 was 1, whereas the fusion decision of cluster 9 was 0. The BS’s location was (500,500). An energy-efficient path in between cluster 48 and the BS was calculated, and detection-related information was sent to the BS. The energy-efficient path was 23-152-206-1-169-22. Similarly, an energy-efficient path in between cluster 9 and BS was calculated, and detection-related information was sent to the BS. An energy-efficient path in between cluster 48 and BS was calculated, and detection-related information was sent to the BS. This energy-efficient path was 6-88-194-227-105-240. The next actual location of the object was (1.1550, 349.7520), and the predicted location was (1.1560, 349.7250). According to the predicted location, the nodes 45, 3, 8 and 11 were awakened for the next round. The predicted location was not in the sensing range of any of the existing clusters. Thus, an on-demand cluster was created, the cluster head of which was node 23. The members of the on-demand cluster were nodes 45, 3, 8 and 11. The BS calculated the location of the object in the first round using the location of nodes 2, 133 and 37. The object location calculated at the BS was (1.0082, 350.0000). This process was continued, and the object’s trajectory was tracked.

#### 4.2.2. Network Coverage and Object Detection

The static clustered network, in which nodes do not change their position after deployment, is energy efficient in the sense that energy wastage due to frequent movement of the mobile nodes according to the object trajectory can be avoided. Two of the factors that affect the object detection accuracy is the network coverage and connectivity. Other factors that affect the object detection accuracy are noise and attenuation. The noise and attenuation were kept constant in this study. We only used mobile nodes to fill the coverage hole and to maximize the network coverage and connectivity. By choosing a mixture of static and mobile nodes for deployment, we could make the system cost effective and hence user friendly. In the articles studied in the literature, a high level of detection accuracy is achieved with a high level of node density, which has an adverse effect on the cost of the system and energy level of the network as the number of communications increases with an increase in the number of nodes, which leads to more energy wastage. We assume that nodes with high power are costlier than low-power nodes. We wanted to minimize the deployment cost by minimizing the number of nodes and by using more low-power nodes. In our study, we tried to achieve a high level of detection accuracy with minimum hardware costs by reducing the number of nodes for deployment. The number of static and mobile nodes used in the EEODTF given in Table 4 was after node optimization using PSO. Table 10 shows the network coverage and detection accuracy for varying area sizes. For network coverage calculation, the point coverage method was used, and for the calculation of detection accuracy, Equation (26) was used.

(26) $$DetectionAccuracy=\left(\frac{CD}{TS}\right)\times 100$$

where *CD* is the number of object positions correctly detected, and *TS* is the total number of steps.

In this study, it was found that there is a directly proportional relationship between network coverage and detection accuracy in the absence of noise and attenuation. However, in the presence of white noise and attenuation, the detection accuracy is lower than the network coverage; see Table 10. The detection accuracy is better in the case of the EEODTF in comparison to the other two models. It is clear that the number of misses increases with the increase in the size of the AOI.

#### 4.2.3. Node Localization Accuracy and Object Localization Accuracy

In this subsection, we studied the effect of node localization accuracy on the object localization accuracy when the range-free method of object localization is used. When the node localization error is lower (i.e., the node localization accuracy is higher), the object localization accuracy is higher. The average node localization error was calculated using Equation (27).

(27) $$\mathrm{Average}\_{\mathrm{Error}}_{\mathrm{NodeLocalization}}=\frac{1}{n}\sum_{i,j=1}^{n}\sqrt{\left({\left(x_{i}-x_{j}\right)}^{2}+{\left(y_{i}-y_{j}\right)}^{2}\right)}$$

where (x_i_,y_i_) is the actual node location and (x_j_,y_j_) is the calculated node location. If the $A\mathrm{verage}\_{\mathrm{Error}}_{\mathrm{NodeLocalization}}$ is less than the tolerance level (the value taken here is 0.02), then the object is treated as being accurately localized. The node localization accuracy was calculated using Equation (28).

(28) $$Accuracy_{nodeLocalization}=\left(\frac{No.\;\;of\;nodes\;correctly\;localized}{Total\;no.\;of\;nodes}\right)\times 100$$

In a similar way, the object localization error and object localization accuracy were calculated.Here, the tolerance value of 0.02 is also taken. If the difference between the predicted value and estimated value is less than the tolerance level, then the object is treated as being correctly localized. This difference was calculated using the Euclidean distance formula.

Table 11 shows the node localization accuracy and object localization accuracy for different area sizes for the EEODTF. The object localization accuracy decreased with an increase in area size. The object localization accuracy was lower than the node localization accuracy. The object localization accuracy decreased when the node localization accuracy decreased.

#### 4.2.4. Energy Consumption and Object Localization Error

In this study, energy consumption during object detection and tracking was monitored, and the result was compared with EETLO and PSOEETTM. The energy consumption calculation was based on three parameters: energy consumed in mobile node movement, energy consumed in object detection and energy consumed in data reporting. Table 12 shows information regarding the energy consumption and object localization error.

The energy consumed by the proposed framework was calculated using Equation (29).

(29) $$E_{\mathrm{consumed}}=\sum_{i=1}^{m}E_{\mathrm{mobileNodeMovement}}+\sum_{j=1}^{t}E_{\mathrm{detection}}+\sum_{k=1}^{t}E_{\mathrm{dataReportingtoCH}}+\sum_{l=1}^{t}E_{\mathrm{dataReportingtoBS}}$$

where m is the number of mobile nodes relocated to increase the network coverage, t is the time taken in the simulation in rounds, E_mobileNodeMovement_ is the energy consumed in relocating mobile nodes, E_detection_ is the energy consumed in the detection of the object, E_dataReportingtoCH_ is the energy consumed by nodes to send detection information to the CH and E_dataReportingtoBS_ is the energy consumed by CHs to report detection-related information to the BS. To report data to the BS, an energy-efficient path was used, which was calculated using Algorithm 7.

**Figure 7:**
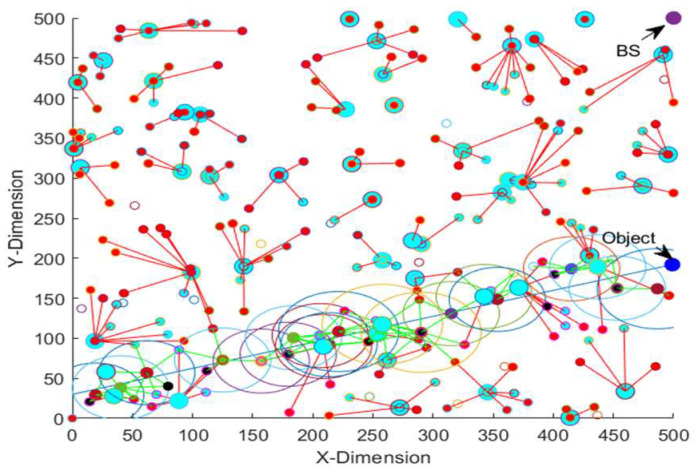
Object in trajectory 2.

**Figure 8:**
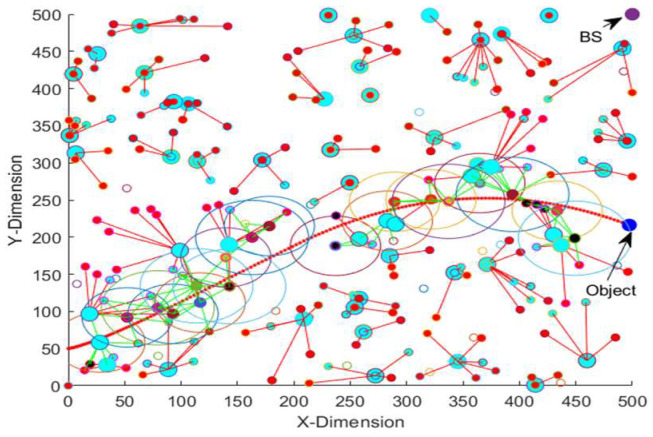
Object in trajectory 3.

Table 13 shows the energy consumption, average detection error and average object localization error of the EEODTF for different object trajectories for the area size of 500 × 500 m^2^. Trajectory 1 is given in Figure 6. Trajectory 2 and trajectory 3 are shown in Figure 7 and Figure 8. Trajectory 2 had the velocity of [2.31, 0.65] and a time step of 0.5. The object moved in this trajectory at a constant speed. In trajectory 3, the object travelled with varying velocity and a time step of 0.5. The velocity was incremented by 0.1 (in both the x-direction and y-direction) when the object’s position was less than 100. However, when the object position was greater than 200, the velocity was decremented by 0.1 in the y-direction, and there was no change in the velocity in the x-direction.

**Figure 9:**
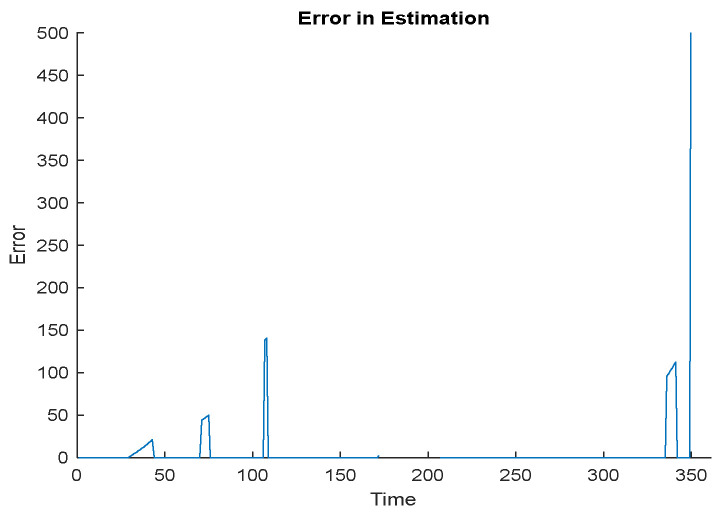
Object localization error in EEODTF for trajectory 1.

Figure 9 shows the object localization error for each step in the trajectory in the case of the EEODTF for an area size of 500 × 500 m^2^.

#### 4.2.5. Network Lifetime Analysis

**Figure 10:**
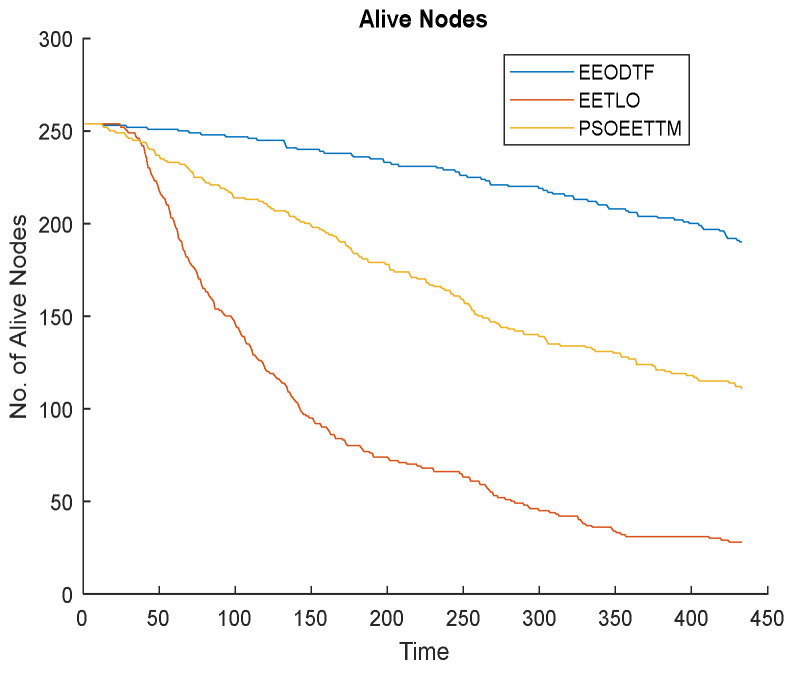
Number of alive nodes vs. time.

**Figure 11:**
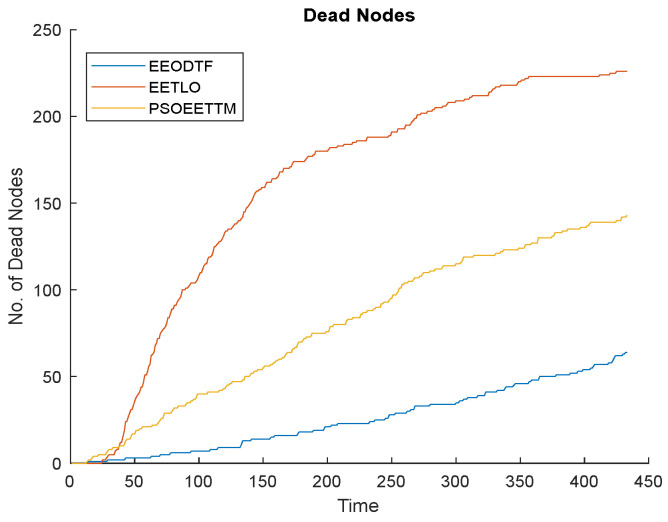
Number of dead nodes vs. time.

**Figure 12:**
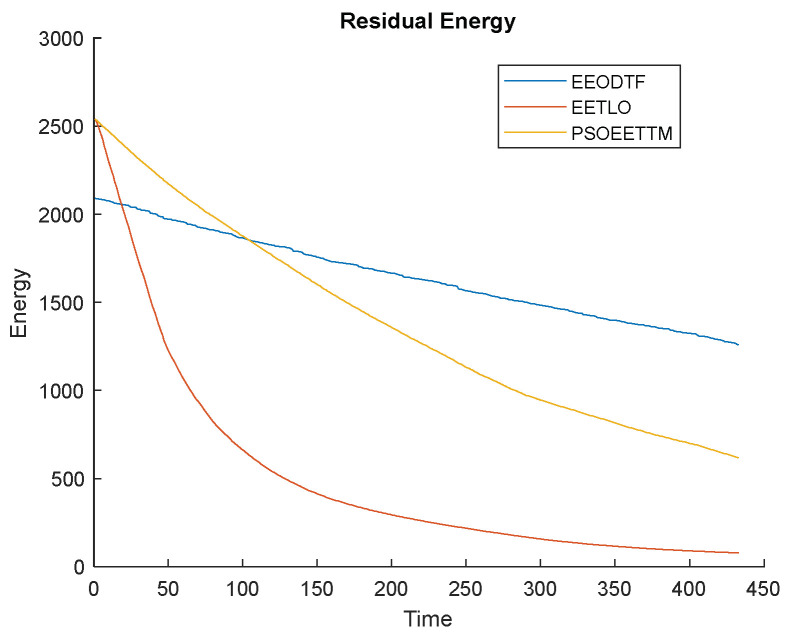
Residual energy of network vs. time.

For any object detection and tracking application, the lifetime of the network is very important. The following figures (Figure 10, Figure 11 and Figure 12) compare the lifetime of the network of the proposed framework, the EEODTF, with the EETLO model and PSOEETTM in terms of the number of alive nodes, the number of dead nodes and the residual energy of the network at the end of the simulation (433 rounds). From the figures, it is clear that the proposed framework is more energy efficient in comparison to the other two models; see Figure 10 and Figure 11. A total of 254 nodes were deployed. For the EEODTF, this was the optimal number of nodes required to cover the AOI. This number of nodes remained the same in the other two models to compare their performance. There were more alive nodes at the end of 433 rounds in the EEODTF than in the other models.

The initial energy value in the EEODTF was lower in comparison to the other two models; see Figure 12. However, at the end there was more residual energy in the proposed framework. This is because the predictive node awakening process was carried out by the BS to track and limit the movement of the mobile nodes. There was less initial energy in case of the EEODTF because low-power static nodes and ordinary mobile nodes were used for deployment.

The time complexity of algorithm 1 is *O(nm),* where *n* is the number of steps travelled by the object at time *t* and *m* is the number of nodes deployed in the network.

## 5. Conclusions and Future Work

The lifetime and quality of service of WSN-based object detection and tracking applications depend on the lifetime of the network. However, the main limitation of WSNs is limited energy, and in order to achieve its purpose, energy efficiency must be maintained during object detection and tracking. In this paper, we proposed an energy-efficient object detection and tracking framework, the EEODTF, which was shown to reduce energy consumption during object tracking and thereby increase the lifetime of the network while not affecting the object detection and localization accuracy. The framework consists of a node deployment algorithm, node localization algorithm, cluster formation algorithm, object detection algorithm, object localization algorithm and energy-efficient routing algorithm for the reporting of data to the BS; see Table 14. This was created in an attempt to reduce energy consumption while carrying out the designated task. Node deployment was optimized using the PSO algorithm to achieve energy efficiency and the maximum coverage of the AOI. Node localization was optimized using the hybrid DV- HOP_PSO_GWO algorithm to reduce the level of object localization errors. Cluster formation, object detection and data reporting processes were optimized using the PSO algorithm, the prediction mechanism and the Firefly algorithm, respectively, for energy efficiency. The performance of the framework was compared with the EETLO and PSOEETTM models using different metrics such as residual energy, the number of alive nodes, the number of dead nodes and detection accuracy. It was found that the proposed framework performed better in comparison to the abovementioned models. Our future work will focus on using a probabilistic sensing model instead of a binary sensing model. We will also focus on the environmental characteristics that affect object detection operations. We will also extend our work to multiple object tracking and object detection applications with adaptive thresholds in the AOI.

## Figures and Tables

**Table 1:** Features incorporated in different models and in our framework.

Paper	Node Optimization	Mobile Node Use	Node Localization	Cluster Structure	Object Detection	Decision Fusion	Route Optimization of Mobile Node	Route Optimization of Packets	On-Demand Cluster
[4]	×	√	×	√	P1	×	√	×	×
[5]	×	×	×	√	P3	×	P4	×	√
[6]	×	×	×	×	P2	×	P4	×	×
[7]	×	×	×	√	P2	×	P4	×	×
[8]	×	×	×	×	P3	×	P4	×	×
[9]	×	×	×	×	P3	×	P4	√	×
[10]	×	×	×	√	P2	√	P4	×	×
[11]	×	×	√	×	P3	×	P4	×	×
[12]	×	×	×	√	P1	×	P4	×	×
[13]	×	×	√	×	P1	√	P4	×	×
[14]	×	×	×	×	P1	√	P4	√	×
[15]	×	×	×	√	P1	√	P4	√	√
[16]	×	×	√	√	P1	×	P4	√	×
[17]	×	√	×	×	P1	×	√	×	×
[18]	×	×	×	√	P1	×	×	×	×
[19]	×	×	×	√	P1	×	P4	√	×
[20]	×	×	×	√	P2	×	×	×	√
[21]	×	×	×	√	P1	×	×	×	×
[22]	×	√	×	√	P1	×	P4	×	×
[23]	×	×	×	×	P1	×	×	√	×
Proposed	√	√	√	√	P1	√	√	√	√

P1—prediction based, P2—probabilistic based, P3—not mentioned, P4—not applicable.

**Table 2:** Comparative Study of Past Research-I.

Attribute	[4]	[5]	[6]	[7]	[8]	[9]	[10]	[11]	[12]	[13]	[14]	[15]	[16]	[17]	[18]	[19]	[20]	[21]	[22]	[23]
Node Mobility	Y	N	N	N	N	N	N	Y	N	N	N	N	N	Y	N	N	N	N	N	N
Sink Mobility	N	N	N	N	N	N	N	N	Y	N	Y	N	N	N	N	N	N	N	N	N
Power Management	G	G	3	G	4	G	G	G	G	G	G	G	G	G	G	G	4	G	G	G
Network Lifetime	G	G	3	G	VG	G	G	G	G	G	G	G	G	G	G	G	VG	G	G	G
Scalability	N	N	N	N	N	Y	N	Y	N	N	N	N	N	N	N	N	N	N	N	Y
Prediction	L	L	L	NL	NL	L	NL	NL	NL	NL	L	NL	L	L	NL	NL	NL	NL	NL	NL
Data Aggregation	G	G	3	N	G	G	G	G	G	G	G	G	G	G	G	G	G	G	N	G
Network Architecture	C	C	3	F	3	T	C	C	C	T	C	C	T	C	3	C	C	C	C	F
Sensing Model	P	3	P	P	3	B	P	P	P	B	B	P	B	B	P	P	P	P	P	P
Static/ Dynamic	S	D	S	D	S	S	D	D	S,D	S	D	S	S	S	S	S	S	D	S	S
No. of Targets	1	1	1	1	2	2	2	2	1	1	1	2	2	1	1	2	1	1	1	1
Target Type	CT	CT	DI	CT	CT	CT	DI	CT	CT	CT	CT	CT	CT	CT	CT	CT	CT	CT	D	CT
No. of Sinks	1	1	1	1	1	1	1	1	1	1	1	1	2	1	1	1	1	1	1	1

C-Cluster, F-Face, T-Tree, B-Binary Sensing Model, P-Probabilistic Sensing Model, S-Static, D-Dynamic, 2-Multiple, CT-Continuous, DI-Discrete, G-Good, NS-Not Supported, VG-Very Good, Y-Yes, N-No, L-Linear, NL-Non-linear, 4-Not Required, 3-Not Mentioned.

**Table 3:** Comparative Study of Past Research-II.

Paper	Advantage	Limitation
[4]	High detection accuracy, energy efficiency	Requires predefined object trajectory, limited energy efficiency, no attention on node density
[5]	Avoids energy wastage by online cluster maintenance and high detection accuracy	Poor sensor scheduling, no attention on node density, difficulties in boundary node selection, no attention on full coverage of AOI
[6]	High object detection accuracy by using probabilistic sensing model	Limited energy efficiency, no attention on node density, no attention on full coverage of AOI and object localization accuracy
[7]	Energy efficiency, reduction in nodes for object tracking	No guarantee of accurate object detection, no attention on node density, no attention on full coverage of AOI
[8]	High object detection accuracy	No guarantee on accuracy of collected data, limited network lifetime and no attention on system cost
[9]	Better tracking accuracy, scalable, minimum network overhead	Increased transmission, no attention on node density
[10]	Better tracking accuracy	Limited energy efficiency, no attention on node density and object localization accuracy
[11]	Accurate cattle detection and recovery	Limited energy efficiency
[12]	Energy efficiency and adjustment in object localization error	Limited energy efficiency, no attention on node density and detection accuracy
[13]	Minimize the object localization error and tolerant to byzantine attack	Limited energy efficiency, no attention on node density, efficiency of model depends on the proper weight determination for each node which is a difficult task.
[14]	Usage of mobile sink, minimum delay in detection, minimum packet loss	Increase in network overhead, no attention on node density, limited energy efficiency
[15]	Minimization in computational cost, optimization in sleep scheduling and cluster formation	Limited energy efficiency, no focus on controlling node density
[16]	Energy efficient tracking, optimization of data reporting	Requires predefined object trajectory, limited energy efficiency, no attention on controlling node density
[17]	Better detection accuracy and energy efficiency	Limited energy efficiency, limited detection accuracy, requires predefined object trajectory, no attention on node density
[18]	Energy efficient sensor scheduling	Limited target detection accuracy and energy efficiency, no attention on node density
[19]	Ability to compensate false detection and missed detection, energy efficient node scheduling	Silent about node density, limited energy efficiency and tracking accuracy
[20]	No energy issue, collaborative target tracking, energy efficient sensor scheduling	Silent about node density, limited detection accuracy and object localization accuracy
[21]	Energy efficiency, minimization of processing burden on nodes, minimization of overhead	No attention on node density and object localization error, cluster formation based on tracking anchor information is a challenge, limited energy efficiency.
[22]	Distributed object tracking, tracking error adjustment	Sensor survival prediction is a challenge, limited network lifetime and no attention on node density
[23]	Consensus-based object detection and localization, considers the case of detection in case of communication link failure	No attention on node density and application lifetime, increased node-to-node communication

**Table 4:** Node Parameters.

Name of the Model	No. of Nodes with Power			Sensing Range of Nodes	
	Static Node	Mobile Node		Static Node and Low-Power Mobile Node (in m)	High-Power Mobile Node (in m)
		Low Power	High Power		
EEODTF	115 (5J)	57 (5J)	82 (10J)	30	40
EETLO	169 (10J)	0	85 (10J)	33.2283	33.2283
PSOEETTM	254 (10J)	0	0	33.2283	0

**Table 5:** Energy parameters and message size.

Parameter	Value
ETX	50 × 0.000000001
ERX	50 × 0.000000001
EFS	10 × 10^−12^
EMP	0.0013 × 10^−12^
EDA	5 × 0.000000001
msgsize	2000

**Table 6:** Parameters used by PSO algorithm in node deployment.

Parameter	Value
C_1_=C_2_	2
w	[0.4, 0.9]
v	0.1 × InitialPosition
No. of Swarms	100
Coverage Degree	1

**Table 7:** Parameters used by node localization algorithm.

Parameter	Value
C_1_=C_2_	2
w	0.5
No. of Swarms	100
v	0.1 × InitialPosition
a	2 × (1 − (itr/maxitr)
maxitr	500
Beacon amount	0.08 × 254

**Table 8:** Simulation parameters used in object trajectory and object detection modeling.

Parameter	Value
vel	[2.31, −0.55]
dt	0.5
Initial_Position	[0, 350]
Noise Variance	0.66
Noise Mean	0
Attenuation Factor	2
Reference Distance(d_0_)	30 m
Reference Signal Strength (S_0_)	−80 dB

**Table 9:** Parameters used in Firefly-based data reporting algorithm.

Parameter	Value
Node Priority	[1, 200]
α	1.0
β	1.0
γ	0.01
θ	0.97

**Table 10:** Network coverage and detection accuracy for varying area sizes.

Area Size (in m^2^)	Name of the Model	Network Coverage	Detection Accuracy	True Positive	Number of Misses
500 × 500	EEODTF	97.6828	96.76	419	14
	EETLO	97.56	72.51	314	119
	PSOEETTM	98.3012	84.2956	365	68
550 × 500	EEODTF	97.7073	93.5010	446	31
	EETLO	96.6084	72.9559	348	129
	PSOEETTM	97.3451	76.9392	367	110
600 × 600	EEODTF	97.0611	77.1154	401	119
	EETLO	94.2553	70	364	156
	PSOEETTM	93.0231	71.3462	371	149

**Table 11:** Node localization accuracy and object localization accuracy.

Area Size (in m^2^)	Total Nodes	Nodes with Correctly Known Locations	Node Localization Accuracy	Total Steps in Object Trajectory	No. of Steps Which Are Correctly Estimated	Object Localization Accuracy
500 × 500	254	197	78.5591	433	339	78.2910
550 × 500	276	216	78.2608	477	367	76.9392
600 × 600	300	226	75.3333	520	371	71.3462

**Table 12:** Average object localization error and energy consumption for AOI of 500 × 500 m^2^.

Models	Average Object Localization Error	Energy Consumed
EEODTF	89.3789	829.7607 J
EETLO	128.5434	2.3146 × 10^3^ J
PSOEETTM	403.3012	1.2178 × 10^3^

**Table 13:** Energy consumption, average detection error and average object localization error.

Trajectory No.	Energy Consumption (in J)	Average Detection Error	Average Object Localization Error
Trajectory 1	829.7607	0.032	89.3789
Trajectory 2	829.4836	0.217	63.6053
Trajectory 3	953.7882	0.256	74.0773

**Table 14:** Summary of Proposed Framework.

Specification and Tools	Description
Specification	The proposed framework is mainly divided into four different parts: node deployment, node localization, object detection and object tracking. Node deployment: Node density and random deployment of heterogeneous mobile and static nodes are considered for full coverage of AOI. Node Localization: Minimize the node localization error to minimize object localization error. Object Detection: Decision fusion with triangulation mechanism is used for energy-efficient object detection and localization. Object Tracking: Energy-efficient data reporting with prediction mechanism is used for error-free tracking. If required, on-demand cluster is created at the cluster boundary to increase the detection accuracy.
Methodology	Our study is on how to achieve energy-efficient tracking in energy-constrained WSNs without affecting the object localization accuracy. This study is completely a simulation-based study. We have considered the case of sensors that cannot be recharged once deployed. Energy management of sensor nodes is done at different levels, such as: limiting the mobile node movement, forming clusters, minimizing the number of nodes engaged for object detection and localization, minimizing retransmission of packets by using energy efficient paths for data reporting. We think that overall energy consumption can be minimized with a collective approach of energy minimization. Simulated object trajectory is used for analysis.
Tools Used	A laptop with 16GB RAM, x64-based processor, a 64-bit operating system, Windows 10 operating system and Matlab R2020b.
Statistical Data	First 10 records of object trajectory 1 are: Sl. ObjX ObjY 1. 0.0 350.0000 2. 1.1550 349.7250 3. 2.3100 349.4500 4. 3.4650 349.1750 5. 4.6200 348.9000 6. 5.7750 348.6250 7. 6.9300 348.3500 8. 8.0850 348.0750 9. 9.2400 347.8000 10. 10.3950 347.5250

## Data Availability

Not applicable.
